# An Ionic Liquid-Assisted Mixed Micelle-Mediated Centrifuge-Less Cloud Point Extraction Spectrophotometric Method for the Determination of Molybdenum(VI)

**DOI:** 10.3390/ijms26104597

**Published:** 2025-05-11

**Authors:** Vidka Divarova, Andrea Gajdošová, Petya Racheva, Kiril Gavazov

**Affiliations:** 1 Department of Chemical Sciences, Faculty of Pharmacy, Medical University of Plovdiv, 120 Buxton Bros Str., 4004 Plovdiv, Bulgaria; vidka.divarova@mu-plovdiv.bg (V.D.); petya.racheva@mu-plovdiv.bg (P.R.); 2 Department of Analytical Chemistry, Faculty of Science, Pavol Jozef Šafárik University in Košice, SK-04154 Košice, Slovakia; andrea.gajdosova@student.upjs.sk

**Keywords:** molybdenum(VI), 4-nitrocatechol, cloud point extraction, spectrophotometric determination, mixed micelle-mediated system, ionic liquid, green analytical chemistry

## Abstract

A novel method for the spectrophotometric determination of trace amounts of molybdenum has been developed. This method utilizes a centrifuge-less cloud point extraction (CL-CPE) in a mixed micellar (MM) system containing a nonionic surfactant (Triton X-114) and an ionic liquid (Aliquat^®^ 336, A336). The chromophore chelating reagent employed was 4-nitrocatechol (4NC, H_2_L). This work marks its first application as a CPE reagent. Under the optimal conditions, Mo(VI) forms a yellow ternary complex with 4NC and A336, which can be represented by the formula (A336^+^)_2_[MoO_2_L_2_]. The method possesses the following characteristics: limit of detection (LOD) of 3.2 ng mL^−1^, linear range of 10.8–580 ng/mL, absorption maximum of 435 nm, molar absorptivity coefficient of 3.34 × 10^5^ L mol^−1^ cm^−1^, and Sandell’s sensitivity of 0.29 ng cm^−2^. The method has been successfully employed for the determination of molybdenum in reference standard steel samples, bottled mineral water, and a molybdenum-containing dietary supplement.

## 1. Introduction

Molybdenum is the sole element of the second transition series that is essential to life. It functions as a cofactor for a variety of enzymes, some of which are particularly important for humans [1]. The synthesis and action of these enzymes are contingent on dietary intake. Molybdenum is naturally present in various foodstuffs, including legumes (lentils, beans, peas), whole grains (oats, barley, wheat), nuts (almonds, peanuts), leafy vegetables, dairy products, and organ meats (liver, kidney) [2]. More than one-third of freshwater supplies contain molybdenum, and some estimates suggest that up to about 20% of the body’s molybdenum intake comes from drinking water [3]. The European Food Safety Authority (EFSA) has proposed an adequate intake (AI) of 65 μg day^−1^ for adults, including pregnant and breastfeeding women, and AIs ranging from 10 to 65 μg day^−1^ for infants, children, and teenagers [4]. The essentiality and toxicity of molybdenum have been addressed many times. Molybdenum is known to be toxic at high concentrations. There have been documented cases of acute poisoning resulting from the ingestion of molybdenum supplements or in the context of occupational activities [2,5]. However, it should be noted that there are dietary supplement manufacturers that offer products for long-term use with Mo(VI) concentrations that exceed the AIs by more than 500%.

Molybdenum-containing materials are of critical importance across a wide range of industries due to their remarkable properties, including high strength, corrosion resistance, and thermal stability. These materials find primary employment in the aerospace, defense, power generation, electronics, chemical, and metallurgical industries. Presently, the predominant application of molybdenum is in the manufacturing of steels and alloys [6]. Molybdenum enhances the hardness, strength, ductility, and resistance of these products, even at high temperatures [7,8].

A variety of techniques have been used to determine molybdenum, including inductively coupled plasma mass spectrometry (ICP-MS), inductively coupled plasma optical emission spectrometry (ICP-OES), graphite furnace atomic absorption spectrometry (GFAAS), X-ray fluorescence spectrometry (XRF), neutron activation analysis (NAA), and spectrophotometry (UV–Vis). Among these techniques, spectrophotometry stands out for its simplicity, cost-effectiveness, convenience, and well-established analytical capability. Its integration with extraction methodologies has been demonstrated to enhance analytical performance [9,10].

**Figure 1:**
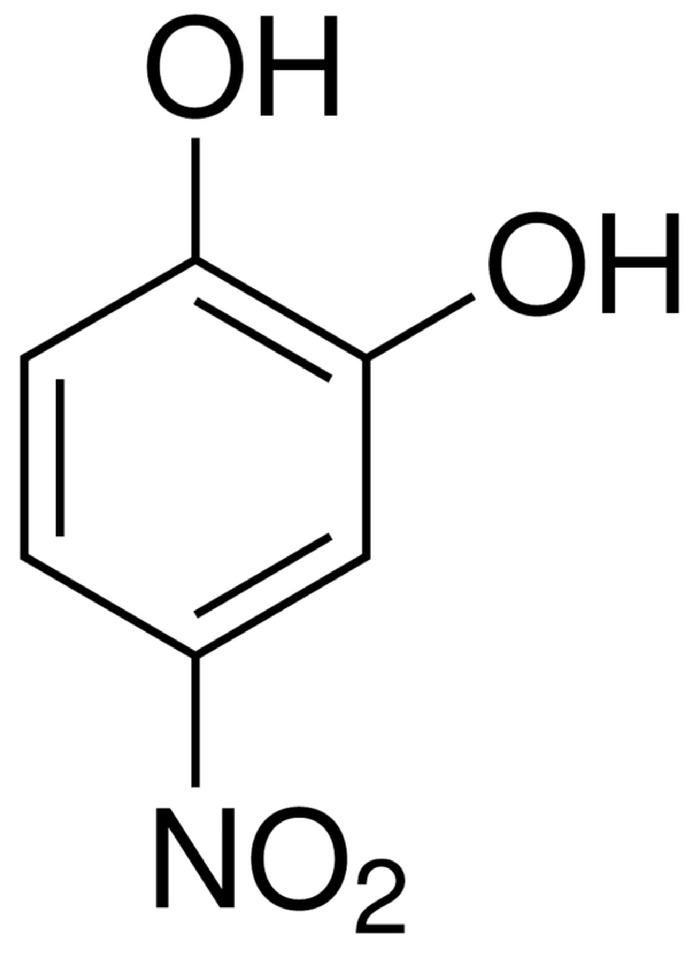
Structure of 4-nitrocatechol (1,2-dihydroxy-4-nitrobenzene, 4NC).

In recent years, a variety of color-forming reagents have been employed for the spectrophotometric determination of molybdenum. These reagents include pyrogallol red [11,12,13], bromopyrogallol red [14], 6,7-dihydroxy-2,4-diphenylbenzopyrylium salts [15,16], and 4-nitrocatechol [17,18,19]. The reagent 4-nitrocatechol (4NC, Figure 1) is a commercially available and inexpensive compound that has been listed by IUPAC as one of the most useful organic analytical reagents [20]. According to European Union Regulation (EC) No. 1272/2008 (CLP regulation), it is classified as a non-hazardous substance [21], meaning it is considered safe for both human health and the environment. However, its use has so far been limited only to applications in classical liquid–liquid extraction (LLE) [22,23,24,25] and electrochemistry [25,26]. The predominant LLE methods for the determination of Mo(VI) involve the use of cationic ion-association reagents of the spherical type (tetrazolium salts), as classified by Tôei [27]. A notable drawback of these methods is their dependence on toxic organic solvents (chloroform and dichloroethane), which are of considerable concern. Our attempts to eliminate these solvents by using cloud point extraction (CPE) as a green analytical technique [28,29,30,31,32,33,34] were unsuccessful, probably because the tetrazolium ion-associates are unstable in CPE processes [35]. However, our preliminary studies have shown that the substitution of tetrazolium salts by chain-type ion-association reagents (such as cetylpyridinium chloride and Aliquat 336) allows for the use of CPE, including its simplest variant, centrifuge-less CPE (CL-CPE).

The objective of the present study is to develop a spectrophotometric method for determining molybdenum in real samples in a mixed micellar (MM) CPE system containing 4NC and both the non-ionic surfactant Triton X-114 (TX-114) and the cationic surfactant Aliquat 336 (A336), which is also an ionic liquid (IL). The employment of MM-CPE systems is regarded as a novel and increasingly significant approach to enhancing the efficacy of CPE [33,34]. A parallel assertion can be made about the utilization of ILs [34].

A336 is a type of IL that contains a mixture of methyltrialkyl(C8,C10)ammonium chlorides [36]. In the fields of analytical chemistry and hydrometallurgy, it functions as an ion-pairing reagent, extractant, and extractor carrier [37,38,39]. Its application in CPE has been documented [40,41], and in a particular study, it was utilized in conjunction with TX-114 [41].

This study marks the inaugural application of 4NC as a CPE reagent. A key element of the study is the ability to directly compare CPE and LLE. Despite the popular belief that CPE is simply a green alternative to LLE that does not use toxic organic solvents, there is a lack of knowledge regarding the differences between the two techniques, particularly in scenarios involving the extraction of ion-association complexes [35].

## 2. Results and Discussion

### 2.1. CPE–Spectrophotometric Optimization

#### 2.1.1. Absorption Spectrum

**Figure 2:**
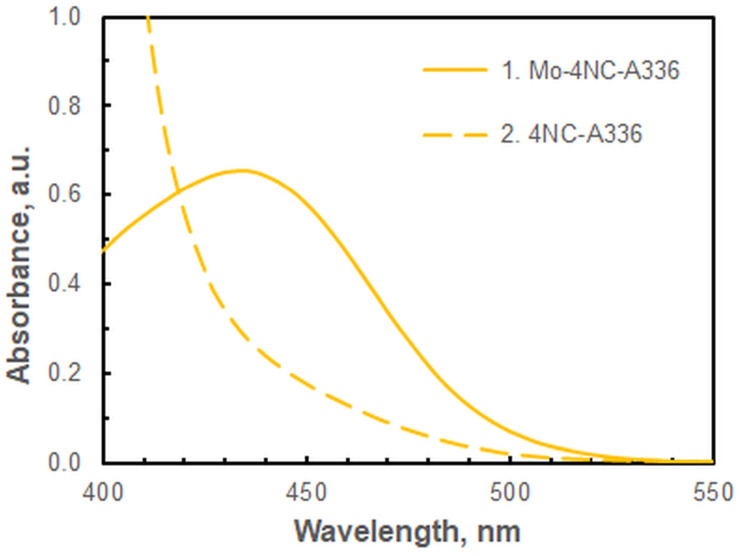
Spectra of the ternary complex against blank (1) and the corresponding blank against water (2): *c*_Mo_ = 2 × 10^−6^ mol dm^−3^, *c*_4NC_ = 3.75 × 10^−4^ mol dm^−3^, *c*_A336_ = 2 × 10^−4^ mol dm^−3^, pH = 5, *V*_buffer_ = 2.0 mL, *w*_TX-114_ = 0.5%, *t* = 55 min at 60 °C, *m*_SRP_ = 3 g.

The absorption spectrum of the Mo-4NC-A336 complex exhibits a maximum at 435 nm (Figure 2). A slight hypsochromic shift is observed in comparison to the maxima of the chloroform-extracted ternary complexes of Mo(VI)–4NC with other cationic ion-association reagents (439–445 nm) [17,18,19]. The underlying causes of this shift may be attributable to solvent effects, the influence of the cationic constituent of the complex, and/or differing stoichiometry. The absorbance at λ_max_ (435 nm) is stable over time. It does not change for at least 1–2 h. By this metric, the CPE system studied outperforms the LLE systems described in previous reports [17,18,19]. Although the absorbance of the blank at 435 nm is relatively high, it is not recommended to perform measurements at higher wavelengths. This would unduly reduce the sensitivity of the determination.

#### 2.1.2. The Effect of pH and Volume of the Buffer

**Figure 3:**
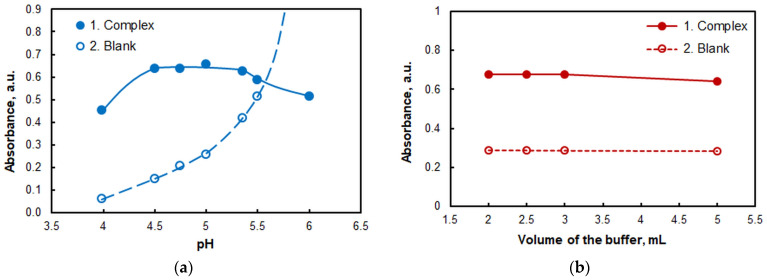
(**a**) Effect of pH: *c*_Mo(VI)_ = 2.0 × 10^−6^, *c*_4NC_ = 3.75 × 10^−4^ mol L^−1^, *c*_A336_ = 2 × 10^−4^ mol L^−1^, *w*_TX-114_ = 0.5%, *V*_buffer_ = 2.0 mL, *t* = 55 min at 60 °C, *m*_SRP_ = 3 g, λ = 435 nm. (**b**) Effect of buffer volume: pH = 5, *c*_Mo(VI)_ = 2.0 × 10^−6^, *c*_4NC_ = 3.75 × 10^−4^ mol L^−1^, *c*_A336_ = 2 × 10^−4^ mol L^−1^, *w*_TX-114_ = 0.5%, *t* = 55 min at 60 °C, *m*_SRP_ = 3 g, λ = 435 nm.

The effect of pH on the absorbance is displayed in Figure 3a. A series of buffer solutions, prepared from 2 mol L^−1^ CH_3_COOH and NH_4_OH, were used to adjust the pH. The analytical signal appears to be maximal over a relatively wide pH range (from 4.5 to 5.4). The buffer is adequate for this range since the p*K*_a_ of acetic acid is 4.75. Therefore, it has a substantial buffering capacity, which effectively manages acidic solutions and thus facilitates sample preparation.

The effect of buffer volume was also investigated (Figure 3b). The absorbance was constant for volumes between 2 and 3 mL. A slight decrease in its value is observed at higher buffer volumes.

#### 2.1.3. The Effect of Reagent Concentrations

**Figure 4:**
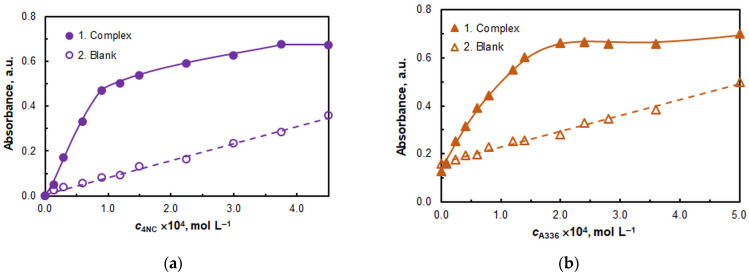
(**a**) Effect of 4NC concentration. *c*_Mo(VI)_ = 2.0 × 10^−6^, pH = 5, *c*_A336_ = 2 × 10^−4^ mol L^−1^, *w*_TX-114_ = 0.5%, *V*_buffer_ = 2.0 mL, *t* = 55 min at 60 °C, *m*_SRP_ = 3 g, λ = 435 nm. (**b**) Effect of A336 concentration: *c*_Mo(VI)_ = 2.0 × 10^−6^, pH = 5, *c*_4NC_ = 3.75 × 10^−4^ mol L^−1^, *w*_TX-114_ = 0.5%, *t* = 55 min at 60 °C, *m*_SRP_ = 3 g, λ = 435 nm.

The effect of reagents concentrations is demonstrated in Figure 4. Subsequent studies were conducted at *c*_NC_ = 3.75 × 10^−4^ mol L^−1^ (see Figure 4a) and *c*_A336_ = 2 × 10^−4^ mol L^−1^ (see Figure 4b). It is noteworthy that at *c*_A336_ = 0, the absorbance is not zero. At the optimal A336 concentration (2 × 10^−4^ mol L^−1^), the increase in the measured signal is over 500% higher than in the absence of A336.

#### 2.1.4. The Effect of TX-114 Mass Fraction

**Figure 5:**
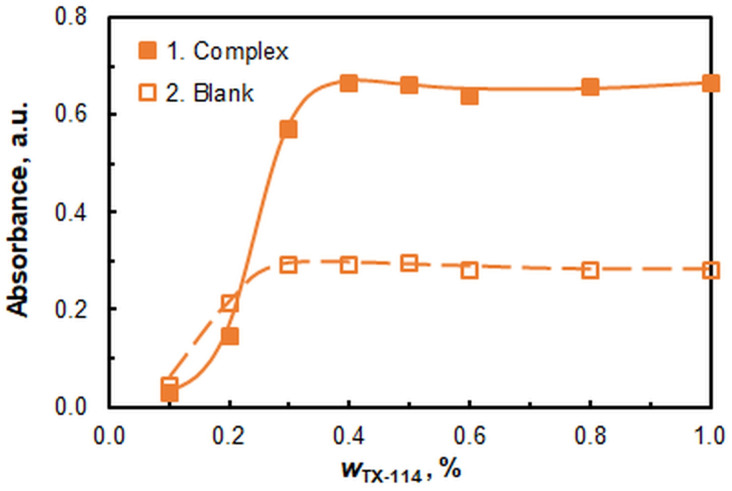
Effect of TX-114 mass fraction. *c*_Mo(VI)_ = 2.0 × 10^−6^, pH = 5, *c*_4NC_ = 3.75 × 10^−4^ mol L^−1^, *c*_A336_ = 2 × 10^−4^ mol L^−1^, *V*_buffer_ = 2.0 mL, *t* = 55 min at 60 °C, *m*_SRP_ = 3 g, λ = 435 nm.

Figure 5 shows the effect of the mass fraction of the non-ionic surfactant on the absorbance. The curve is characterized by a steep left part and a well-defined plateau. It is evident that saturation is attained at a mass fraction of approximately 0.4%. No significant drop in the extraction efficiency is observed at higher *w*_TX-114_ values, as is the case with other MM-CPE systems [11,42,43,44]. Further studies were performed at a *w*_TX-114_ of 0.5%, which corresponds to a mass ratio of the two surfactants close to 1:58 (A336:TX-114). The low absorbance of the sample at *w*_TX-114_ below 0.3% can be explained by difficulties in the formation of the surfactant-rich phase (SRP) under the experimental CL-CPE conditions.

#### 2.1.5. The Effect of Incubation Time

**Figure 6:**
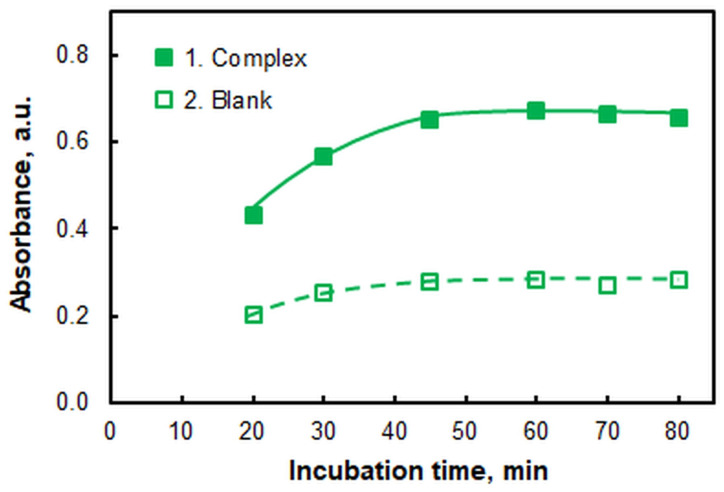
Effect of incubation time at 60 °C. *c*_Mo(VI)_ = 2.0 × 10^−6^, pH = 5, *c*_4NC_ = 3.75 × 10^−4^ mol L^−1^, *c*_A336_ = 2 × 10^−4^ mol L^−1^, *w*_TX-114_ = 0.5%, *V*_buffer_ = 2.0 mL, *m*_SRP_ = 3 g, λ = 435 nm.

Figure 6 shows the effect of residence time at a water bath temperature (*t*) of 60 °C. The time was counted from the moment the samples were immersed in the heated water. The recommended incubation time deduced from Figure 6 is 55 min.

#### 2.1.6. The Cooling Time Experiments

The cooling process that facilitates phase separation can be conducted in a refrigerator at a temperature of approximately −20 °C for a duration of 45–60 min [45,46]. A shorter cooling time may result in an insufficiently viscous surfactant reach phase (SRP), which can impede the efficacy of phase separation. Cooling for more than 1 h may cause freezing of the supernatant and further delay.

#### 2.1.7. SRP Processing

The upper aqueous phase following the cooling process was separated by decantation. The viscosity of the resulting SRP was reduced by the addition of 0.5 mL of ethanol [45]. The final solution, which was subsequently analyzed, was obtained by carefully adding water to a final mass of 3.00 g. The mixture (SRP + ethanol + water) can be readily homogenized by shaking, a process that is less time- and effort-consuming than the ethanol-free procedures [46].

A summary of the optimized parameters is shown in Table 1.

### 2.2. Stoichiometry, Formula of the Extracted Complex, and Equation of Complexation

In aqueous media, Mo(VI) forms anionic complexes with 4NC (H_2_L), which can be represented by the formulas [MoO_2_(OH)_2_L]^2−^ and [MoO_2_L_2_]^2−^ [47]. In the presence of cationic ion-association reagents and organic solvents, neutral ternary complexes of both types have been reported [17,18,19]. Which of the above anionic species is stabilized depends on the experimental conditions and the nature of the cationic reagent.

**Figure 7:**
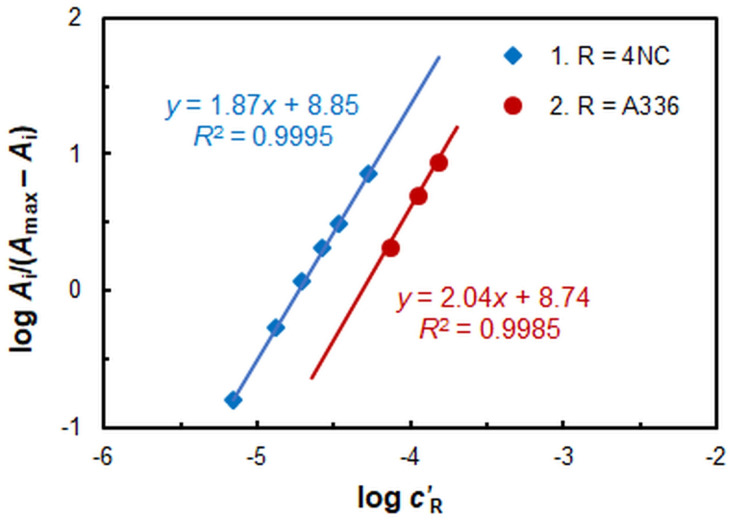
Application of the mobile equilibrium method for the determination of the 4NC-to-Mo (1) and A336-to-Mo (2) molar ratios.

**Figure 8:**
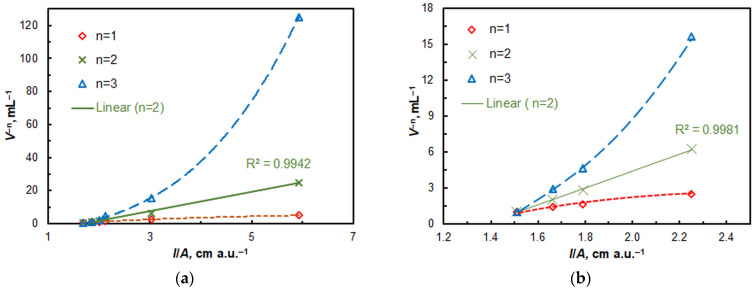
Application of the straight-line method of Asmus for the determination of the 4NC-to-Mo (**a**) and A336-to-Mo (**b**) molar ratios.

The molar 4NC:Mo and A336:Mo ratios in the CPE system were determined by the mobile equilibrium method [48] (Figure 7) and the straight-line method of Asmus [49] (Figure 8). The results obtained suggest that under optimal conditions, the complex composition is 1:2:2 (Mo:4NC:A336). Consequently, its formula is (A336^+^)_2_[MoO_2_L_2_]. The stabilization of this complex is favored by maintaining a relatively high pH (pH_opt_ = 4.75) at which deprotonation of 4NC is facilitated. The equation of complex formation can be expressed as follows: 2 HL^−^ + MoO_4_^2−^ + 2 A336^+^ ≡ (A336)_2_[MoO_2_L_2_] + 2 OH^−^.

### 2.3. The Constant of Extraction

The conditional extraction constant characterizing the CPE of the complex was calculated by two methods based on the A336 saturation profile curve (Figure 4b): the mobile equilibrium method [48] and the Holme–Langmyhr method [50]. The results can be found in Table 2.

### 2.4. Beer’s Law and Analytical Characteristics

The relationship between light absorption and Mo(VI) concentration was investigated under optimal conditions (see Table 1). The study revealed a satisfactory linearity up to 580 ng mL^−1^ Mo(VI) (*R*^2^ = 0.9991, *n* = 10). The linear regression equation was determined to be *A* = 3.48*γ* + 0.001, where *γ* denotes the mass concentration (μg mL^−1^). The standard deviations (SDs) of the slope and intercept were determined to be 0.04 and 0.01, respectively. The apparent molar absorption coefficient was found to be 3.34 × 10^5^ L mol^−1^ cm^−1^, and the Sandell sensitivity was 0.29 ng cm^−2^. The limit of detection (LOD) and quantitation (LOQ), calculated as 3 and 10 times the SD of the blank divided by the slope, were 3.2 ng mL^−1^ and 10.8 ng mL^−1^, respectively.

The preconcentration factor, calculated as the ratio of the volume of the sample (50 mL) to the volume of the final solution (3 g ≈ 3.05 mL), was 16.4. A similar value of 16.8 was determined by dividing the slope of the calibration line obtained after CPE by the slope obtained without CPE (i.e., without TX-114 and heating). The absence of a significant disparity between these values suggests that the complex formation process occurring in aqueous media remains unaltered during the CPE process.

### 2.5. The Effect of Foreign Ions

The effects of various foreign ions on the determination of Mo(VI) are demonstrated in Table 3. The presence of significant amounts of SO_4_^2−^, NO_3_^−^, Cl^−^, Br^−^, alkali ions, alkaline earth ions, Ni(II), and Zn(II) is tolerable. The most significant interfering factors are Al(III), Cr(III), Cu(II), Mn(II), V(V), and W(VI). Sodium fluoride appears to be an effective masking agent for Al(III). At the working conditions, Cr(III) forms an intensely colored complex with 4NC and A336 (*λ*_max_ ≈ 475 nm). However, this complex is susceptible to disruption in the presence of Na_2_EDTA. The same is true for the complexes Cu(II) (*λ*_max_ = 451 nm), Fe(III), V(V), and Mn(II). It is anticipated that the selectivity will be elevated in media with higher acidity. Therefore, the determination can be performed at a pH of 4.5, which is the lower limit of the pH interval in which the maximum absorption is observed (see Figure 3).

### 2.6. Analytical Application

The developed procedure was implemented for the analysis of two reference standard steel samples (Table 4). The relative standard deviations (RSDs) for these determinations were ≤3.3%.

Commercial mineral water from two Bulgarian brands (Gorna Banya and Devin) was also analyzed. The results are presented in Table 5.

The panel of analyses was expanded through the incorporation of a dietary supplement, Molybdenum Drops—Concentrate for Long-Term Use (Extract Pharma LTD, Sofia, Bulgaria) [51]. According to the manufacturer, each drop contains 50 μg Mo(VI). To verify this claim, two kinds of experiments were planned. They were designed to consider both the averaging of results from several drops and the analysis of a single drop.

Experiment 1: An aqueous solution containing seven drops of the pharmaceutical product (the daily dose recommended by the manufacturer for adults) was prepared. The analytical result obtained (*n* = 4; ±SD) was 362 ± 12. Consequently, the molybdenum content in one drop was approximately 51.7 μg.

Experiment 2: Three parallel solutions were prepared, with each solution containing a single drop of the preparation (the recommended daily dose for children aged 3 to 10 years). The mass of each drop was initially determined using a balance (*m*_1_ = 0.0554 g, *m*_2_ = 0.0658 g, and *m*_3_ = 0.0604 g). The analysis demonstrated that the Mo(VI) content of the three samples differed significantly (43.8 ± 2.2 μg, 59.8 ± 0.88 μg, and 51.0 ± 0.5 μg Mo) due to the variation in drop masses. However, the mean mass value of 51.6 μg Mo(VI) closely corresponds to the outcomes of Experiment 1.

### 2.7. Comparison with Other Extractive Methods

A comparative analysis of the present method’s characteristics with those of other extractive methods for molybdenum determination is provided in Table 6. The present spectrophotometric method is characterized by simplicity, cost-effectiveness, convenience, and ecological friendliness. The use of a centrifuge is not necessary because phase separation occurs spontaneously. The separation of the two phases is easy and convenient (by decantation) and does not require the use of a syringe or micropipette [52,53]. The addition of a salting-out agent, as is common in [11,12,14], is also not required. The reagents utilized are readily available in the commercial sector and do not necessitate synthesis, a notable advantage over the procedures [15,16]. Finally, the method is robust and reliable, as evidenced by the large optimal ranges of the parameters studied. In this respect, it successfully competes with many other methods [11,14,15,53].

In contrast to the LLE methods described in [17,18,19], the present method can be classified as environmentally friendly and safe for laboratory personnel. In addition, it is about 6 to 14 times more sensitive mainly due to the higher phase volume ratio (16.4 vs. 2 or 1). Since the complexation takes place in a less acidic environment (compared to the LLE methods mentioned above), some disadvantages can be noted: higher blank absorbance and higher RSD.

**Figure 9:**
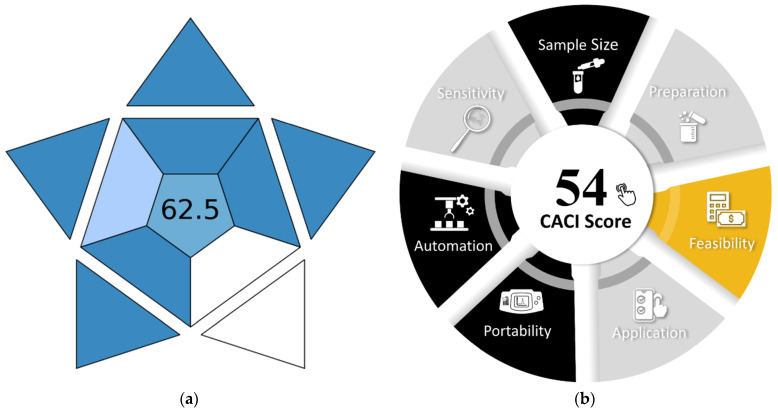
Application of the Blue Applicability Grade Index (BAGI) (**a**) and the Click Analytical Chemistry Index (CACI) (**b**) to evaluate the proposed procedure.

Figure 9 presents an evaluation of the proposed method by two recently developed green and analytical performance metric tools: the Blue Applicability Grade Index (BAGI) [54] and the Click Analytical Chemistry Index (CACI) [55]. The scores obtained from the two metrics (greater than 60 in the first case and greater than 50 in the second) characterize the novel approach as “practical”.

**Table 6:** Comparison with other extractive methods for molybdenum determination.

Reagent (s)	Procedure	Detection	Surfactant (s)	pH	Sample	Linear Range/ ng mL^−1^	LOD, ng mL^−1^	λ_max_, nm	10^−4^*ε,* L mol^−1^ cm^−1^	Ref.
PR	MM-CPE	UV–Vis	TX-114 + CTAB	4.6	River water and coastal waters	1.23–37.0	1.24	608	NR	[11]
DHDPhB + NaSal	RT-CPE	UV–Vis	TX-100	1.8	Water and milk	7.9–160	2.3	560	NR	[15]
DHMPhB + NaSal	RT-CPE	UV–Vis	TX-100	2.0	Water, rose hips, and pharmaceuticals	160–1800	50	530	NR	[16]
Nile blue A + Oxalate	UTA-CPE	FAAS	PONPE 7.5	4.5	Milk, vegetables, and foodstuffs	3–340	0.86	–	–	[53]
VPB + KSCN	MM-CPE	FAAS	TX-114 + CPC	2.0	Beverages and foodstuffs	7.5–1800	2.18	543	–	[44]
8-HQ	CPE	FAAS	TX-114	4.5	Mineral water	NR	40	–	–	[52]
QA	CPE	GFAAS	TX-114	3.6	Seawater and tap water	0.03–0.6	0.007	545	–	[56]
BPR + KI	CPE	UV–Vis	CTAB	1	Steel and water	0.3–320	0.1	576	NR	[14]
4NC + BTC	LLE	UV–Vis	–	1.8–4.0	Steel and ferromolybdenum	200–6700	NR	445	2.38	[18]
4NC + BZC	LLE	UV–Vis	–	1.4	Synthetic mixtures, steel, and water	18.6–3100	5.6	439	5.5	[17]
4NC	IL-MM-CL-CPE	UV–Vis	TX-114 + A336	4.5	Mineral water, steel, and a food supplement	10.8–580	3.2	435	33.4	This work

Abbreviations: 4NC, 4-nitocatechol; 8-HQ, 8-hydroxyquinoline; A336, Aliquat 336; BPR, bromopyrogallol red; BTC, Bluetetrazolium chloride; BZC, Benzalconium chloride; CPC, cetylpyridinium chloride; CPE, cloud point extraction; CTAB, cetyltrimethylammonium bromide; DHDPhB, 6,7-dihydroxy-2,4-diphenylbenzopyrylium salt; FAAS, flame atomic absorption spectrometry; GFAAS, graphite furnace atomic absorption spectroscopy; IL-MM-CL-CPE, ionic liquid-assisted mixed micelle-mediated centrifuge-less CPE; LLE, liquid–liquid extraction; MM-CPE, mixed micelle-mediated CPE; NaSal, sodium salicylate; NR, not reported; PONPE 7.5, polyethyleneglycolmono-p-nonyphenylether; PR, pyrogallol red; RT-CPE, room temperature CPE; TX-100, Triton X-100; TX-114, Triton X-114; QA, Quinalizarine; UTA-CPE, ultrasonic-thermostatic-assisted CPE; UV–Vis, spectrophotometry; VPB, Victoria pure blue BO.

## 3. Materials and Methods

### 3.1. Reagents and Chemicals

The Mo(VI) solution (2 × 10^−4^ mol L^−1^) was prepared by means of the dissolution of (NH_4_)_6_Mo_7_O_24_·4H_2_O (cryst. extra pure, Merck, Schnelldorf, Germany) in water. The 4NC was procured from Fluka AG (Buchs, Switzerland, >98%). The concentration of the prepared aqueous solution was 1.875 × 10^−2^ mol L^−1^. The other chemicals utilized as aqueous solutions were Na_2_EDTA·2H_2_O (Fillab EOOD, Plovdiv, Bulgaria, >99.5%; *c* = 0.1 mol L^−1^) and TX-114 (laboratory grade, Merck, Schnelldorf, Germany; *w* = 10%). The ionic liquid A336 was procured from Merck (Schnelldorf, Germany) and dissolved in methanol (*c* = 1 × 10^−2^ mol L^−1^). The calculations were performed using a molar mass of 432 g mol^−1^, which is consistent with the value reported in Ref. [36]. Buffer solutions were prepared by combining appropriate volumes of 2 mol L^−1^ aqueous solutions of ammonia and acetic acid. Deionized water (18.2 MΩ cm) or distilled water was utilized during the course of the experiments.

### 3.2. Instrumentation

A UV–Vis spectrophotometer (Ultrospec 3300 pro; Garforth, UK) equipped with 10-mm cuvettes was utilized to conduct spectrophotometric investigations. The pH was estimated using a WTW InoLab 7110 pH meter (Weilheim, Germany). The samples were subjected to heating in a GFL 1023 water bath (Berlin, Germany). An Ohaus Pioneer PA214C analytical balance (Parsippany, NJ, USA) was employed for mass quantification.

### 3.3. Samples and Sample Preparation

Bottled mineral water was procured from a local supermarket and analyzed the following day. For the analysis, 30 mL aliquots were utilized.

The food additive, Molybdenum Drops (Extract Pharma^®^, Sofia, Bulgaria), was supplied by an online pharmacy. According to the manufacturer, each drop of the preparation contains 50 μg Mo in the form of sodium molybdate. The formulation also comprises potassium sorbate and vegetable glycerin. The prepared aqueous solutions had volumes of 50 mL (7 drops) and 25 mL (1 drop).

The steels (approximately 0.5 g each) were prepared for analysis using a two-stage procedure that was a combination of methodologies described in the literature: stage 1 [17,57,58] and stage 2 [18]. The objective of the initial stage was the separation of tungsten(VI). In the subsequent stage, iron and other undesirable elements were precipitated. To mask residual amounts of these elements, Na_2_EDTA (0.1 mL of a 0.1 mol L^−1^ solution) was utilized.

### 3.4. Optimization Procedure

Solutions of TX-114, ammonium acetate buffer, Mo(VI), 4NC, and A336 were sequentially transferred to pre-weighed 50 mL conical centrifuge tubes. The mixtures were then diluted to 50 mL with water, shaken to ensure homogeneity, and heated in a water bath at 60 °C for a specified time. The tubes were then briefly cooled under running water and stored in a freezer set at −20 °C for approximately 45–60 min. This was done to complete the precipitation process and make the top layer easy to remove by decanting. Then, 0.5 mL of ethanol and drops of water were added to the viscous SRP to give a total mass of 3.00 g. Finally, the solutions were homogenized by shaking and transferred to cuvettes for measurement of light absorption.

### 3.5. Recommended Procedure for the Determination of Mo(VI)

Transfer an aliquot of the analyzed solution (10.8–580 ng mL^−1^ Mo) into a 50 mL conical centrifuge tube of known mass. If necessary (e.g., for steel analysis), add 0.1 mL Na_2_EDTA solution (0.1 mol L^−1^). Then add 2.5 mL of a 10% TX-114 solution, 2.5 mL of a buffer with pH 4.5, 1.0 mL of a 1.875 × 10^−2^ mol L^−1^ solution of 4NC, and 1.0 mL of a 1.0 × 10^−2^ mol L^−1^ solution of A336. Dilute the sample with water to the specified volume (50 mL) and heat in a 60 °C water bath for 55 min. After cooling in a freezer set at −20 °C for 55 ± 5 min, remove the top layer of the sample (the surfactant-poor phase) by decanting. Carefully add 0.5 mL ethanol and a few drops of water to the SRP to give a total mass of 3.00 g. Homogenize the mixture by gentle shaking and transfer it to the spectrophotometer cuvette. Measure the absorbance at 435 nm against a blank. Calculate the unknown molybdenum concentration from the calibration curve.

## 4. Conclusions

A novel method, designated as IL-MM-CL-CPE, has been developed for the spectrophotometric determination of Mo(VI). The efficacy of the method was demonstrated through analyses of real samples and the application of two green and analytical performance metric tools. The method has been shown to be simple, cost-effective, reliable, sensitive, feasible, and practically acceptable. Notably, the employment of 4NC in a CPE application represents a noteworthy advancement, as it is the first time this reagent has been utilized in this context. The findings of the conducted studies, particularly those addressing the impact of foreign ions, indicate that 4NC (an inexpensive and non-hazardous reagent according to the CPL regulation) has the potential to serve as a foundation for the development of a series of environmentally friendly CPE methods for the determination of other metal ions (e.g., Cr(III) and Cu(II)).

## Figures and Tables

**Table 1:** Optimal CPE–spectrophotometric conditions.

Parameter	Optimal Value/Range	Figure
Wavelength, nm	435	Figure 2
pH	4.5–5.0	Figure 3a
Volume of the buffer, mL	2–3	Figure 3b
Concentration of 4NC, mol L^−1^	3.75 × 10^−4^	Figure 4a
Concentration of A336, mol L^−1^	2.0 × 10^−4^	Figure 4b
Mass fraction of TX-114	0.4–0.5	Figure 5
Incubation time at 60 °C, min	55	Figure 6
Refrigeration time at −20 °C, min	55	–
Test tube capacity, mL	50	–
Mass * of the final solution, g	3	–

* In the presence of 0.5 mL ethanol.

**Table 2:** The logarithmic value of the conditional extraction constant.

Method	Log *K*_ex_ *
Holme–Langmyhr method	8.8 ± 0.4
Mobile equilibrium method	8.6 ± 0.3

* ± Standard deviation (SD).

**Table 3:** Determination of Mo(VI) (7.7 μg) in the presence of foreign ions.

Ion	Salt Formula	Ion:Mo(VI) Mass Ratio	Mo(VI) Found, μg	*R*, %
Al(III)	Al_2_(SO_4_)_3_·18H_2_O	1 ^a^ 100 ^b^	7.7 7.9	100 102
Ba(II)	BaCl_2_	1000	7.3	95.6
Br^−^	NaBr	2000 ^c^	7.8	101
Ca(II)	Ca(NO_3_)_2_	750	7.7	100
Cd(II)	CdCl_2_	100	7.4	96.2
Cl^−^	NaCl	2000 ^c^	7.3	95.2
Cr(III)	Cr_2_(SO_4_)_3_	2 ^a^	8.0	104
Cr(VI)	K_2_CrO_4_	2	7.9	103
Co(II)	CoSO_4_·7H_2_O	10	7.6	99.4
Cu(II)	Cu(SO_4_)_2_·5H_2_O	5 ^a^	7.6	98.6
EDTA^2−^	Na_2_EDTA·2H_2_O	500 1000	7.4 6.8	96.2 88.0
F^−^	NaF	200 500	7.9 8.1	102 105
Fe(III)	Fe_2_(SO_4_)_3_	5 ^a^	7.5	97.9
HPO_4_^2−^	Na_2_HPO_4_·12H_2_O	100	7.9	103
Li^+^	Li_2_SO_4_·H_2_O	1000	8.2	107
Mg(II)	MgSO_4_·7H_2_O	2500 ^c^	7.9	103
Mn(II)	MnSO_4_·H_2_O	5 ^a^	7.3	95.6
Ni(II)	NiSO_4_·7H_2_O	1000 ^c^	7.8	102
NO_3_^−^	NaNO_3_	2000 ^c^	7.7	99.4
Pb(II)	Pb(NO_3_)_2_	10 ^c^	7.9	103
Re(VII)	NH_4_ReO_4_	100	8.2	106
SO_4_^2−^	MgSO_4_·7H_2_O	10,000 ^b^	7.9	103
V(V)	NH_4_VO_3_	2 ^a^	8.0	104
W(VI)	Na_2_WO_4_·2H_2_O	1	8.5	111
Zn(II)	ZnSO_4_·7H_2_O	1000 ^c^	7.9	102

^a^ In the presence of Na_2_EDTA·2H_2_O (3.8 mg). ^b^ In the presence of F^−^ (3.8 mg). ^c^ Higher mass ratios were not studied.

**Table 4:** Determination of Mo in referent standard steel samples (*n* = 4).

#	Certified Mo Content, %	Other Ingredients, %	Mo Found,* %	*R*, %
1	0.96	17.7 (W), 4.71 (Co), 4.21 (Cr), 1.58 (V), 0.35 (Mn), 0.18 (Si), 0.081(C), and the balance Fe	0.964 ± 0.032	100.4
2	0.97	11.7 (W), 4.09 (Cr), 0.35 (Mn), 0.12 (Ni), 0.10 (Cu), 0.083 (C), 0.22 (Si), and the balance Fe	0.960 ± 0.040	99.0

* ±SD.

**Table 5:** Determination of Mo(VI) in unspiked and spiked samples of bottled mineral water (*n* = 3).

Bottled Water	Mo(VI) Concentration, ng mL^−1^		RSD, %	*R*, %
	Added	Found ^c^		
Gorna Banya ^a^	0	16.7 ± 1.5	9.0	–
	19.2	35.8 ± 2.7	7.7	99.6
	38.4	57.4 ± 4.1	7.2	106
	57.6	74.1 ± 9.2	12.4	99.7
	76.8	92.4 ± 5.6	6.0	98.7
Devin ^b^	0	21.8 ± 1.8	8.9	–
	19.2	40.0 ± 3.2	8.1	94.6
	38.4	62.4 ± 2.2	3.6	106
	57.6	81.7 ± 1.0	1.2	104
	76.8	96.0 ± 0.8	0.8	96.7

^a^ Mineral water from borehole #4 (Gorna Banya) and Domus Ravine spring. ^b^ Mineral water from boreholes #5 and #3, Devin field. ^c^ ±SD.

## Data Availability

Data are contained within the article.
